# Clinical Duties

**Published:** 1885-10

**Authors:** P. P. Wells

**Affiliations:** Brooklyn, N. Y.


### HOMŒOPATHIC PHYSICIAN,

A MONTHLY JOURNAL OF MEDICAL SCIENCE.

"If our school ever gives up the strict inductive method of Hahnemann, we are lost, and deserve only to be mentioned as a caricature in the history of medicine."—CONSTANTINE HERING.

Vol. V. OCTOBER, 1885. No. 10.

### CLINICAL DUTIES.—WHAT THEY ARE, AND HOW THEY ARE TO BE PERFORMED.

P. P. WELLS, M. D., BROOKLYN, N. Y.

[Read before I. H. A., June, 1885.]

They are those of the doctor beside the sick-bed, when he is about to attempt a cure. These duties all have their objective in what is expressed by this one word. What, from this standpoint, then, is the first duty of the healer? The answer will be determined by the views he takes of the nature of that with which he is about to attempt to deal. Among healers there are two classes who hold very different opinions as to this nature. One looks on sicknesses as material entities, *things*, each with its particular form, nature, and name—a something distinct from the patient it has invaded, and which is to be expelled by material agencies more or less violent in their action, and this is to be proportioned to the severity of the attack—the more severe this, the more potent is to be the violence required for its expulsion. Sicknesses are named and called *diseases*, the nomenclature being determined by certain generic phenomena found in each member of the class, and this naming is called the *diagnosis* of the case, which is the *first* effort of the materialist prescriber. Having, from these phenomena, found his name, he uses them for his next step, making them suggestives of a supposed nature of the attack and of the internal condition of part or parts which this invader has caused, and this he calls the *pathology* of his case, which, having settled to his satisfaction, he is prepared to enter on his next duty—that of finding the means of cure. And here is the circle in which the materialist prescriber always and in all cases moves. The circle is complete when he has decided on the means to be employed. And this decision is controlled by the views the prescriber entertains of the imaginary internal condition of his patient's internal organs—his so-called pathology. He *imagines*, again, that it is in the nature of some material agent or *agents* (for he does not often use his gun single shotted), when brought into contact with these organs, so sick, to bring them relief. And here is the whole circle of the theory and practice of materialistic medicine. The first duty, the *diagnosis;* the second, the *pathology;* the third, to *find* the *curative.* Is not the circle a perfect one, and each step in the practice reasonable? Let us see.

The first step—diseases as things, entities. Are they so, indeed, or has this been assumed? As this has never been and never can be demonstrated, it must be regarded as assumed. And on what foundation does the assumption rest? Evidently on no other than that of the imagination of the materialist himself. Certainly this is no sufficient foundation for a *beginning* of the practical duties of "*scientific medicine.*"

"No other foundation than an imaginary one, after all these centuries of examinations of the bodies of the dead? Have they learned nothing from all these of the essential nature of disease?" Apparently nothing; for the simple reason that this nature could not then, there, and so be learned. "But did not Louis so discover the essential nature of typhoid fever? and was not this disclosed in the changed condition of Peyer's patches and Brunner's glands?" Not more than one could decide by the silt at the delta the length, breadth, depth, and velocity of the stream which deposited it. These anatomico-pathological changes are only the *result* of forces and processes which have produced them, which forces and processes material medicine ignores in its first step of practical duty, and so it has guessed and guessed wrong, and has been industriously trying to back up its false judgments by autopsies which are really voiceless as to all pertaining to this essential nature. They are *results* of sick processes, these changes so discovered, and not the essential diseases, more than is the expectorated matter in lung affections essentially the pneumonia of which it is the product.

And then the second duty of the materialist prescriber—to settle the *pathology* of his case. Has he a better foundation for his conclusions in this than in those of his first step? An intelligent examination of the matter seems to show the two have a common basis. This is in the nature of the case. No man has seen or can see that this internal condition is as his pathology assumes it to be. Only the Creator of the body can see its internal condition during life. The prescriber who proceeds on this as the basis of his clinical duties goes on an assumption of facts which have never been demonstrated and never can be, and oftener than otherwise have no existence except in his own imagination; so that if he finds his "pathology often misleading," as a hearty admirer and advocate of modern pathology has declared that it is, it is only what should have been expected, because in the nature of the thing it could not be otherwise. Even doctors cannot reasonably be expected *always* to guess right. And judging from the practical results of this kind of proceeding, we are sure we do those who thus practice no injustice if we say these often prove them to have guessed very wrong. And yet these men parade these *guesses* as an essential part of "*scientific medicine*"*!* Does the word "*scientific*" characterize only that which *is known?* But this perversion of its use attaches it to that which is *not known* but only *guessed at!* Is it any wonder, then, if this guessing, this so-called pathology, is "often misleading"?*

But how as to the solution of the means or agent for the cure? Is there not something better than guessing to guide the materialist in this choice? Let us see. Having his diagnosis and pathology guessed out, he brings before his mind a long list of classified drugs, which stand each as it may chance to have been classed, either as an emetic, cathartic, narcotic, antispasmodic, etc., etc., to the end of this arbitrary classification. He *guesses* his patient requires the assumed action of some one of these classes, say the emetics, and to him who recognizes only this one effect of all the members of the class it is not very important whether he takes one or another, if only his patient gets a good, satisfactory vomiting. A knowledge of the specific action of the different members of the class on the various other organs of the body and their functions makes no part of his education, nor has this any place in his practical thoughts. He employs the drug for its power to effect vomiting. Beyond this he neither intends nor wishes any result whatever. But will the drug heed his intentions and limit its action to this one organ and to this one of its functions? Will it not rather act

---

*W. S. Searle in "Single Remedy," HOMŒOPATHIC PHYSICIAN, Vol. II, p. 313.

just like itself and all other drugs in all other cases? *i. e.*, will it not go on expending its force on other organs and functions, just as it is its nature to do, wholly regardless of the honest intentions of this well-meaning and guessing prescriber? So we find it here as in his two first steps, only and ever guessing, and always guessing *in the dark.* From beginning to end of his clinical duties he has no guide but his own imagination or that of another, and of this it cannot be denied he makes free and constant use, and this, forsooth, because he has no other resort or resource.

Does failure of success, when so guided, at all weaken his confidence in this his ultimate authority? Let the history of the treatment of the last epidemic of malignant cholera in the city of Naples, in 1884, answer, when it sums up the result in the statement that of every one hundred so then and there treated fifty-three died. Does not the result convict of a practice *in the dark?* And yet this practice, so wholly made up from beginning to end of unmixed *guessing*, is paraded before the world as the embodiment of "*scientific medicine*"! the practical outcome of which, in the late experience of it at Naples, was fifty-three deaths in every hundred treated.*

But we have said there are two classes of opinions as to the nature of that which the prescriber is called upon to cure. We have given one of these, the material, and have seen its make up and the outcome of its practical application. Assuming what cannot be demonstrated as fact, at each step, from diagnosis to death, is it any marvel if fifty-three of the one hundred treated died? And is there any denunciation too severe of the bold impertinence and blind ignorance which parades this guessing and failure before the world not only as the "scientific," but as the *only* "scientific" in practical medicine? Can human impudence and arrogance go farther?

There is another view of this nature, which is founded on the perceptible phenomena of the sickness in hand, and therefore on elements which are knowable and presumably known. Those who entertain this view are neither called on to assume nor guess as to any element of the case with which they have to deal. This view limits their attention strictly and altogether, and in all cases, to facts which are or may be *known.* In this their philosophy and practice are in sharpest contrast with that of

---

*In the same city, in the epidemic of 1854, only one-seventh of one per cent. of those treated specifically (*i. e.*, homœopathically) died!! Number of cases so then and there treated, seven hundred and three.

those who by their different views are so wholly consigned to a practical life of guessing. This other philosophy of sickness goes back to the foundation of its first principles, and there finds the sickness not a thing but only a change in the action of those forces which execute the different functions of the different bodily organs, so that, while before this change, these functions were all performed in that harmonious balance and agreement which conserves the organism in whole and in part, now, by the change induced by the impact of the morbid cause, this harmony is lost, and thereby this action has become distinctive. It maintains, this other philosophy, that this is just what sickness is—a *change in the action of immaterial forces*, and not a material *thing* at all. That it is just this and nothing more. And that, as to its clinical duties, the first step in those of the opposite view—the diagnosis—the name—has no place in the solution of the problem of finding the specific curative. Not that right diagnosis is either unimportant or to be ignored. It is always to be made and made rightly, but never to be otherwise used than in its true province; and this other philosophy places this wholly aside from the problem of finding the true specific. Because not admitted into this most important of clinical duties, it is not to be inferred that the specific prescriber holds true diagnosis in light esteem. It excludes it from this part of clinical duties for the one reason that specific medical philosophy never prescribes for *names*, but ever and only for this change in the action of the life forces. So that again it places this immaterial dynamic nature of sicknesses in contrast with the other, which regards these as things.

Then as to the next step—the pathology of the case—the views taken by these two parties are equally diverse and antagonistic—the one imagining a condition of internal organs and processes, and calling this its pathology; the other gathering all the perceptible phenomena of the sickness, and these, taken as a whole, *are the pathology* it recognizes, and it holds that in these is contained all that can or need be known of the pathology of the case; that these constitute its pathology, and besides these there is and can be no other which can add aught of truth to any man's knowledge.

Then as to selection of the curative, and the views of these two parties as to its action, they are, if possible, still more at variance. The object of the materialist is, in most cases, to excite violent action in the functions and sensations of the organism, hoping for a curative influence in the reaction from this violence, to be gained in some not very well defined way. Thus he seeks relief by indirect means. On the other hand, his opponent seeks the same end, always and only, by the most perfect, direct action of the chosen agent on the affected organ or organs, and on the functions of these, and by this direct action to change these affected organs and functions to their normal healthy state. This, to this second party, is all there is or can be in any cure. And in the use of the specific remedy his object is always to give his medicine in such doses as while the sick organs and functions are affected curatively it leaves all other organs and functions unaffected. This object, as well as avoidance of increase of present sufferings, is generally realized where no more of the specific is employed than is required for the cure.

Then the prerequisite knowledge which shall qualify these two classes to select curing agents, each according to his own philosophy of the phenomena before him, is not less remarkable for the difference in kind and extent than are the differences which we have already noticed as to nature, diagnosis, and object in drug solution and administration. The materialist is content with the knowledge which enables him to place his drug in its proper class. He knows that which he selects will vomit, purge, etc., and he proceeds, on this limited and comparatively unimportant intelligence, to give it to his patient, in doing which he has reference to this one element of its action, being wholly oblivious as to all other effects of his dose, because for the most part he is entirely ignorant of them, and then, if he knew them, his system of prescribing gives him no possible use for them. To him *sicknesses* are *diseases*—things. So he needs *diagnosis*—*i. e.*, a *name* for his *thing;* and his *pathology*, that he may have some kind of notion of the *kind* of *thing* he has before him; and then, does this kind, as he imagines, require vomiting, purging, narcotism, sweating, etc., or to what class of his materia medica shall his *guessing* lead him!

The other, the dynamist we will call him for convenience, begins and proceeds with a sickness he is to cure in a very different manner. To come at its *nature*, and to a knowledge of the agent which shall cure it, he first notes the history of the case as it has been developed previous to his visit. Then all the visible departures from a normal state which the patient presents. Then he questions the sick as to all his discomforts and pains in utmost detail; and of each of these as to the circumstances of its appearance, and as to whatever aggravates or relieves these. How are these affected, if at all, by time of day or night, by position or motion of any kind, or by the action of any function of life, as breathing, eating, defecation, etc.? In what order of succession have these pains, etc., appeared, and what is the character of the pains—are they fixed or moving—if moving, in what direction? Having questioned *all* the functions of the sick body in this manner, and written down the answers, the prescriber has before him all that he or any man can *know* of the *nature* of the sickness he is to cure. He has no need for guessing as to this nature if he have the intelligence requisite to the interpretation of this language of nature, of which he has before him this written example.

If he has this intelligence he has discovered that, as with all sickness not the result of chemical or mechanical causes, his case began by a disturbance of some one or more functions of the bodily organs, and that these functional disturbances *preceded* any change of material tissues which he may now perchance meet in the case before him. From this he may learn the fundamental truth which pertains to all sicknesses, with the exceptions noted above, that these are in the beginning only changed functions, and therefore they are always *dynamic*, and not material, in their intimate nature. They are here and always but a change in the mode of action of the force which controls and executes the bodily functions. This is the first lesson for the specific prescriber to learn. Till he has compassed this, any efforts on his part as a healer must, from the nature of the case, be prosecuted in the fog and obscurity of old school physic. In this dynamic nature are found the first rays of therapeutic light; and in this is the beginning of the radical differences between the material and dynamic schools of medicine, which makes any philosophical coalescence of the two impossible, and any proposed attempt at this only the most absurd of conceivable ideas. It is only to attempt a coalescence of knowledge and ignorance, or of any other impossible incompatibles. And yet this coalescence has been talked of as an event to be expected, "*on a scientific basis.*" This well-sounding expression seems to have so completely filled the ears and brains of some who would be leaders of doctors, that they have no perception of or place for the only therapeutics God has given us, and to which alone pertains aught of "*science.*" It is "science" on the one side and guessing on the other—and can these coalesce?

Here then is the first lesson—the true *nature* of disease, and here is the first clinical duty of him who will obey God's law of healing, to gather before him *all* the elements of the sickness he will cure, not that from these he may infer some peculiar internal condition, but to make them a solid and rational foundation for the right performance of his next duty. With the supposed internal condition, called by the materialist his *pathology*, the dynamist has neither concern nor need. The record before him, as we have supposed it to be, *is* his pathology, and is made up of facts known, and he has no need to supplement these with guessings, his own or another's, and he is only doing a foolish work who attempts this.

Then, as in these two first duties, gaining a knowledge of the essential nature of sicknesses and their pathology, if there are indeed two, the material and dynamic schools start with so different views and pursue their inquiries with so different objectives, that they are no less diverse in the third duty—finding the curative. For this the materialist only needs his catalogue of classified drugs, and his guess that the case, as he has come to understand it, requires some member of some one of these classes, and he selects that one, or those, which he guesses are the proper ones, and he gives them with hope, though in all his guessing, so far, he has had the guidance and aid of no one known, fixed principle, nor of any other thing or consideration than his own whim or imagination.

The dynamist, on the other hand, before he can take the first step in this duty, requires knowledge of the actions of each and every drug, from the total of which he is to select the *one* his case requires, and this as to how each acts on each and every organ and function of the living body. And this knowledge he is to have in a form and gained by a process which has left no place for doubt or guessing. This knowledge has come from experiments and observations, which, for the most part, have been the work of capable men who were fully impressed with a sense of the importance and value of the results of their labors. He is to select the *one* drug, not because of any supposed importance of any one of its effects on any one organ or function of the body of his patient, but because the selected drug is, in its modifications of healthy functions, the most perfect imitator of the sick aberrations before him. Having found this and given it, he has found and given the curative which his case requires, and of this he has the assurance of the law which declares the relationship of sicknesses and curatives. The knowledge of this law is another of the differences of the two schools, one of which recognizes and accepts this as guide and controller of clinical selection of curatives, while the other neither has this nor any other guide than his own fancy in the selections he may make.

There is another radical difference between material and dynamic medicine in the answer to the question—*how much?* It is one of fundamental principle. The materialist answers this question by another—*How much will he bear?*—the idea being that the patient is to have all of the drug he can receive inside of the line, which, if passed, will imperil his life; while, of course, the intention is always to avoid the danger involved in excessive dosing, it not unfrequently happens that life is not only endangered, but lost, from the ignorance of the prescriber of the other actions of the drug than that which has given to it its place in the class from which it has been selected. He is not only ignorant of these other effects of his selected drug, but also and absolutely of the specific relationship between drugs and sick conditions, which exists in the very nature of these two chief factors in the clinical problem, the relationship of similarity, by which an unknown element in the action of the drug agent may unwittingly be brought in contact with a sick condition like to itself, and this may be followed by an aggravation of this element of the sick condition wholly incompatible with continued life. The death which follows in these circumstances is always a mystery to the unfortunate and ignorant doctor and a surprise to friends, who will require, and no doubt receive, a very learned and "scientific" explanation of a fact of which he who explains knows just absolutely nothing at all. The whole matter is to his mind a total darkness, and yet he explains.

The dynamist, on the other hand, meets the question—How much?—ever and in all cases with the reply, "the *least* quantity of medicine to accomplish the desired result."* He knows nothing of good can come to his patient by any addition of drug force to the sum the cure requires. And then, if he be prepared rightly to perform the duties he has undertaken, he is secured from such unpleasant surprises as we have seen his materialist neighbor is liable to by his more perfect knowledge of the multitudinous effects of the action of drugs on the many bodily organs and functions. Then he is still further protected from these by a knowledge of the fact of the almost infinitely greater susceptibility of sick organs and functions to drug actions which are similar to the sick phenomena of his case. "The least possible" is the safety both of the doctor and his patient.

But how is the practitioner who has yet to gain an experience which will enable him to answer just how much this "least possible" is in a given case? Let him be instructed by those whose clinical record has shown the greatest proportion of cures to the cases treated, and whose knowledge of and loyalty to

* Dowling's reply to Professor Palmer.

law have rightfully made them our teachers. It is not, perhaps, too much to expect of such an one that he has been so far educated into the true philosophy of disease as to know that this, in all its phases, is dynamic in its nature. If such an one should say yes—but when this dynamic aberration has by its progress produced results such as large collections of pus or solid tumors, which, by pressure on parts or organs necessary to life, to an extent that this is thereby endangered—how then about the dynamic nature of the problem of cure? The answer is hardly needed, that in so far as this pressure has become a dominant factor in the case, it has thereby been transferred to the category of mechanical cases, which may, and often do, require mechanical means for their relief.

The nature of the sickness to be cured being accepted as dynamic, *i. e.*, existing in the aberrations of the actions of forces which control and execute functions, then the duty of gathering a knowledge of *all* these aberrations before proceeding to consider the choice of a remedy is self-evident, if this choice is to be made under the guidance of the only known law of relationship between curatives and sicknesses, and the only "*science*" of therapeutics founded on this law. This relationship, existing only in the *similarity* of the sick phenomena to these aberrations of functions produced by the action of the drug, it is easily understood that until *all* these phenomena are known, it cannot be known whether the action of any drug is more like these than that of any other, which is just what is to be known *positively* before its selection can be in compliance with the demands of specific prescribing. And just here is where the great difficulty in specific prescribing is met. It is in getting a knowledge of *all* the sick phenomena of the case. This duty is made difficult by the limited intelligence of both the physician and the patient. This difficulty is best known and appreciated by him who has been most loyally faithful in his endeavors to overcome it. However great this may be, it is to be met and overcome, or there is to be no certainty of results in any attempts at specific prescribing.

These preliminary duties having been performed by members of each school of practice, and the remedy to be given by each selected, then the answer to the question—In what *form* shall it be given?—is found to disclose another and important difference in the methods of each. For the best results, as he understands these, and in his endeavors to secure them, the materialist, having accepted sicknesses as *material* in their nature, proceeds to deal with them by the use of material masses of his chosen agent, or agents, in crude form. In this he is but acting in logical accord with his initial error, which accepted disease as a material thing. He meets matter with matter, and as to the selection of the matter he is to employ, he guesses as well as he can. If the result be that fifty per cent. of the sick so treated die, who will say that from his erroneous standpoint and premises he has not guessed as well as he could? Is not the fault in these rather than in the unfortunate man who accepts them, and proceeds to discharge clinical duties on this foundation?

Are we wrong when we affirm that this whole process of this materialist prescriber is but a succession of guesses from the beginning to the end? If this be a just judgment, what measure of indignation does he merit who foists this upon the world and claims for it that it alone is the embodiment of "*scientific* medicine"! And is he receiving any injustice if, with whatever the measure of indignation heaped on him, there be with it an equal sum of contempt poured on his baseless claim? It may be, it is admitted, that this claim for this process of guessing—a process which, whatever else may be in it, is as utterly empty of even the faintest shodow of anything like "*science*" as Satan is of holy purpose—shall be deemed beneath contempt. Is it not full time that this empty, false pretense should be exposed, and those of our own school who affect an imitation of it should receive their merited rebuke?

The dynamist who accepts the immaterial nature of disease is also logical if he meets this by use of his discovered remedy in a dynamic form. But there may be differences of judgment as to how far the process of dynamization shall be carried to best fit the dose for a given case. It is well that right ideas of this process of dynamization should be received. It will save much muddle. The discovery of the process had its origin in an endeavor to diminish the force of the dose of the *legal*—*i. e.*, the *similar*—remedy, by reducing the quantity of drug matter in it. The manner of effecting this gave us an expression of what was supposed to have happened in the process, which is altogether misleading as to the fact. Each step in the reduction came to be termed a "*dilution*" or an "*attenuation*," neither of which describes what has happened as to the *curing power* of the medicament treated. It may be sufficiently correct to say of the *drug matter* that it has been "*attenuated*" or "*diluted*," but to say this of the *dynamic element* of the drug, which cures, is absurd and misleading. To *dilute* or *attenuate* a *force* is not readily conceived of as possible. This force may be *developed* by the process of dynamization, and this is proved by an abundant experience to be just what has happened in preparing his drugs for the use of the dynamist prescriber. This has been so abundantly proved in the experience of the most successful prescribers the world has known, that no more is needed by ingenuous minds than the mention of the fact and the names of those who have thus verified it. It may be known from the experiences of Hahnemann, Hering, Staph, Bœnninghausen, Gross, and Haynel, and by that of a multitude of others less known to fame, by all who are willing to accept truth because it is truth; but it is bad to know that there are those with whom truth has no convincing power. What, indeed, has truth to do with minds which imagine themselves capable of limiting the operation of God's law by their own silly resolves, especially if they can get enough of them together to resolve strong and keep each other in countenance while they do it?

It will be well for the inexperienced practitioner to study the example and record left us by the worthies whose names we have just given. Let him imitate their example as far as is possible for him and to the utmost, and if he fails of a record which equals theirs, he will by so doing make his nearest approach to theirs. Let him individualize his cases as they did theirs, *i. e.*, let him drop from his thoughts, as they did from theirs, all reference to *names*, as of classes of sickness, and bring out and put on record the symptoms which characterize this individual member of the class before him, for it is these which relate his case to its curative. Having done this, let him give his related remedy in a degree of dynamization in the direct ratio of the degree of resemblance found between these symptoms and those his drug has been found to produce when taken by healthy persons, *i. e.*, the greater the number of similar symptoms, and the more perfect the similarity, the higher should be the number of the dynamization employed. He should also, if emulous of the best practical results, dismiss all thoughts of *drug matter* in his doses, and trust his numbered dynamizations as the veritable *potencies* they are, and not as "*dilutions*" or "*attenuations*," which they are not. And further, in this let him remember he is only carrying out the fundamental law of Homœopathy and its corollaries to their logical termination. Having so selected and so given his remedy, let him keep himself free from all nervous anxiety as to the result. Leave the dose to take care of itself and the patient, and only repeat it as the case may require.

There are those who are regarded by some as dynamists in practice who have abandoned the individualization here described and in its place have resorted to the generalization characteristic of materialistic medicine, and in their practical duties are not readily to be distinguished from this by any principles of philosophy or practice. These, having abandoned the method of Hahnemann, should neither be regarded, nor their methods imitated, as those of dynamist healers. They are already, as Hering declared such would be, only caricatures of doctors, and not in any degree healers of men. Their chief function seems to be, after their foolish endeavors to mix practical incompatibles, to manufacture *resolves* for associated bodies which, when passed, have for their chief result *the stultification of both.*

