# Errata

**Published:** 1885-11

**Authors:** 


Errata.—The following errata occur in Dr. Wells' article on "Clinical Duties," in our last issue: P. 335, line 24, and p. 338, line 18, for *solution* read *selection;* p. 337, line 10, for *distinctive* read *destructive;* p. 342, line 18 from bottom, for *in* compliance read *a* compliance.

